# Maternal psychological distress during the COVID-19 pandemic and structural changes of the human fetal brain

**DOI:** 10.1038/s43856-022-00111-w

**Published:** 2022-05-26

**Authors:** Yuan-Chiao Lu, Nickie Andescavage, Yao Wu, Kushal Kapse, Nicole R. Andersen, Jessica Quistorff, Haleema Saeed, Catherine Lopez, Diedtra Henderson, Scott D. Barnett, Gilbert Vezina, David Wessel, Adre du Plessis, Catherine Limperopoulos

**Affiliations:** 1 Developing Brain Institute, Children’s National Hospital, Washington, DC USA; 2 Department of Pediatrics, School of Medicine and Health Sciences, the George Washington University, Washington, DC USA; 3 MedStar Washington Hospital Center, Washington, DC USA; 4 Critical Care Medicine, Children’s National Hospital, Washington, DC USA; 5 Prenatal Pediatrics Institute, Children’s National Hospital, Washington, DC USA

**Keywords:** Brain imaging, Paediatric research, Epidemiology, Stress and resilience

## Abstract

**Background:**

Elevated maternal psychological distress during pregnancy is linked to adverse outcomes in offspring. The potential effects of intensified levels of maternal distress during the COVID-19 pandemic on the developing fetal brain are currently unknown.

**Methods:**

We prospectively enrolled 202 pregnant women: 65 without known COVID-19 exposures during the pandemic who underwent 92 fetal MRI scans, and 137 pre-pandemic controls who had 182 MRI scans. Multi-plane, multi-phase single shot fast spin echo T2-weighted images were acquired on a GE 1.5 T MRI Scanner. Volumes of six brain tissue types were calculated. Cortical folding measures, including brain surface area, local gyrification index, and sulcal depth were determined. At each MRI scan, maternal distress was assessed using validated stress, anxiety, and depression scales. Generalized estimating equations were utilized to compare maternal distress measures, brain volume and cortical folding differences between pandemic and pre-pandemic cohorts.

**Results:**

Stress and depression scores are significantly higher in the pandemic cohort, compared to the pre-pandemic cohort. Fetal white matter, hippocampal, and cerebellar volumes are decreased in the pandemic cohort. Cortical surface area and local gyrification index are also decreased in all four lobes, while sulcal depth is lower in the frontal, parietal, and occipital lobes in the pandemic cohort, indicating delayed brain gyrification.

**Conclusions:**

We report impaired fetal brain growth and delayed cerebral cortical gyrification in COVID-19 pandemic era pregnancies, in the setting of heightened maternal psychological distress. The potential long-term neurodevelopmental consequences of altered fetal brain development in COVID-era pregnancies merit further study.

## Plain language summary

We investigated whether the mental health of pregnant mothers influenced the brain development of their fetuses during the COVID-19 pandemic. We imaged the brains of fetuses before and during the COVID-19 pandemic and asked the mothers questions about any distress experienced during pregnancy. We found increased maternal stress and depression in women pregnant during the pandemic compared to those pregnant before the pandemic. The volume of some areas of the fetal brain decreased, and there was a delay in the development of the brain folds in the fetuses of the distressed mothers.

## Abstract (web summary)

Lu, Andescavage et al. scanned fetal brains by MRI and assessed maternal distress before and during the COVID-19 pandemic. Impaired fetal brain growth, delayed cerebral cortical gyrification and increased maternal distress was seen in COVID-19 pandemic era pregnancies.

## Introduction

Intrauterine programming refers to early developmental responses to environmental exposures that in turn may influence an individual’s lifelong health^1^. The timing, duration, and severity of fetal exposures may adversely impact tissue and organ system development through multiple pathways, including nutrition, oxygen supply, inflammatory changes, dysregulated hormonal exposure, and epigenetic changes^2^. The fetal brain is especially sensitive to such changes, and it is increasingly recognized that developmental and neuropsychiatric conditions manifesting later in life have their origins in the fetal period^3,4^. Several studies have shown that prenatal exposure to maternal psychological distress results in structural and functional changes in brain development of young children through school age, including regional changes in surface area, gray matter and amygdala volumes along with cortical thinning^5–7^. Furthermore, emerging evidence links these structural differences in brain development with neurobehavioral function in children and adolescents^8,9^. However, this body of research also highlights the challenges in distinguishing the effects of prenatal from postnatal exposures with the potential cumulative impact of prolonged exposures across extensive periods of development. Given the impact of not only the presence, but the timing, severity, and duration of adverse prenatal exposures on the developing brain, the ability to precisely characterize fetal brain development represents an advance in the field. Recent studies have demonstrated an association between maternal psychological distress and altered structural and functional development of the fetal brain^10–14^, allowing for an enhanced understanding of prenatal mental health exposures on later neuropsychological function in offspring.

The Severe Acute Respiratory Syndrome Coronavirus-2 (SARS-CoV-2) responsible for COVID-19 (coronavirus disease 2019) pandemic was first identified in Wuhan, China in 2019 and continues to exact widespread morbidity and mortality across the globe^15^. During the pandemic, elevated levels of depression, anxiety, post-traumatic stress and psychological distress^16–18^, have been reported. In pregnant women, concerns around potential fetal COVID-19 exposure, as well as social isolation, food and housing insecurity, unemployment, and inequitable access to health care, play an important role in elevated pregnancy-related psychological distress^19^. Specific to pregnant women, both rates of anxiety and depression have increased, along with heightened symptomology of clinical mental health conditions^20–22^. One early study suggests that prenatal maternal distress during the COVID-19 pandemic may decrease amygdala-prefrontal connectivity in infants at 3 months of age, particularly in settings of lower social support^23^. The psychosocial impact of this pandemic on fetal brain development, however, remains largely under-reported. The objective of this investigation is to determine the effects of maternal mental health on in vivo human fetal brain development during the COVID-19 pandemic. Our overarching hypothesis is that heightened maternal stress, depression, and anxiety during the COVID-19 pandemic will adversely influence fetal brain growth and development, even in the absence of confirmed COVID-19 exposure. Our results show that maternal stress and depression are significantly higher in the pandemic cohort, compared to the pre-pandemic cohort. We also demonstrate decreased regional fetal brain volumes and delayed brain gyrification in the pandemic cohort.

## Methods

### Study participants

This study involved two sequential enrollments: (1) 137 healthy pregnant women from March 2014 to February 2020 (“pre-pandemic”); (2) 65 women without known COVID-19 exposures from June 2020 to April 2021 (“pandemic”) for a fetal brain magnetic resonance imaging (MRI) study from low-risk obstetrical community hospitals in Washington, DC (Supplementary Fig. 1). The first enrollment period was part of a longitudinal study of normative fetal brain development in low-risk obstetric patients, and the second was a natural history observational study of fetal brain development during the COVID-19 pandemic. Study procedures were identical across both enrollment periods. Healthy pregnant volunteers had a normal prenatal history that included normal screening, laboratory, and ultrasound studies. Exclusion criteria were multiple gestation pregnancy, known or suspected congenital infection, syndromic or dysgenetic features in the fetus, documented chromosomal abnormalities, or any maternal contraindication to MRI. Similarly, subjects reporting the use of medications or substances other than prenatal vitamins or supplements were excluded (e.g., prescribed medications, tobacco, marijuana, or alcohol use). Enrolled fetuses found to have structural (encephaloclastic or dysgenetic) brain abnormalities on fetal MRI, or postnatal confirmation of a genetic syndrome were subsequently excluded from the study. Parental education and employment data were collected from each participant during study visits. Following approval by the Institutional Review Board at Children’s National Hospital (Protocol 1373 for the pre-pandemic cohort, approved on January 9, 2011, and Protocol 14257 for the pandemic cohort, approved on May 1, 2020), written informed consent was obtained from all participants.

### Maternal distress

Four well-validated maternal distress measures were completed by each pregnant woman on the day of the MRI, including the Spielberger State Anxiety Inventory (SSAI, range: 20 to 80)^24^, Spielberger Trait Anxiety Inventory (STAI, range: 20 to 80)^24^, Perceived Stress Scale (PSS, range: 0 to 40)^25^, and Edinburgh Postnatal Depression Scale (EPDS, range: 0 to 30)^26^. Values higher than the following thresholds were considered elevated: maternal state anxiety >40, trait anxiety >40, stress > 15 and depression >10^25,27–29^.

### MRI data acquisition

Multi-plane multi-phase single shot fast spin echo (SSFSE) T2-weighted images for fetal brain were acquired on a 1.5 Tesla GE Discovery MR450 scanner (GE Healthcare, Milwaukee, WI, USA) using an eight-channel surface receiver coil (USAI, Aurora, OH). The following acquisition parameters were used: echo time = 160 ms; repetition time = 1100 ms; field of view = 320 × 320 mm^2^; matrix = 256 × 256; 2 mm slice thickness and 50 to 70 consecutive slices for full fetal brain coverage in the axial, coronal, and sagittal planes for a final in-plane resolution of 1.25 × 1.25 mm^2^. Each subject was scanned up to two time points in the fetal period.

### Image processing

Motion correction was first conducted on fetal brain T2-weighted multi-plane images using the slice-to-volume registration method^30^. This procedure reduced interslice motion artifacts and provided images with enhanced contrast and resolution, and coherent anatomic boundaries in 3D space. 3D brain images with severe motion artifacts that affected the ability to distinguish brain tissues such as cortical gray matter (CGM), white matter (WM), lateral ventricles, brainstem and cerebellum were excluded from the analysis. Automatic segmentation of the brain tissues was then implemented using the Developing Brain Region Annotation with Expectation-Maximization (Draw-EM) algorithm^31,32^. Draw-EM utilizes Expectation-Maximization (EM) algorithm to segment a brain into different tissue types as well as detailed structures of the brain^31^. Two sets of tissue labels were generated from Draw-EM: the segmentation file with 9 labels^31^ and the parcellation file with 50 labels^32^. Manual correction of tissue labels of the segmentation and parcellation files was performed by a trained research team member (K.K.), who had more than 5 years of experience using ITK-SNAP in fetal brain segmentation during the time of this work^33^. Fifty-five scans (20%) were randomly chosen and segmented by a second trained examiner (N.R.A.) to evaluate the inter-rater reliability. The intraclass correlation coefficients for all measured regions between the two examiners were higher than 0.95.

Ten brain regions of both the right and left hemispheres were extracted from segmentation and parcellation files (Supplementary Fig. 2): the frontal, parietal, temporal, and occipital lobes, anterior and posterior cingulate gyrus, insula, and corpus callosum were extracted from the parcellation file, and the deep gray matter (DGM) and ventricles from the segmentation file. These brain regions were imported to BrainSuite version 18a to generate 3D surface mesh models^34^. Each mesh model contained a set of 3D coordinates of the surface vertices and a set of triangular mesh. Every surface vertex was associated with one of these 10 brain regions.

### Fetal brain volumes and cortical folding

Brain tissue volumes from the segmentation file were determined based on the voxel sizes of the images, including CGM, WM, DGM, cerebellum, brainstem, and hippocampus (Supplementary Fig. 2d–f)^35^.

To characterize 3D fetal brain morphology, three cortical features, including the surface area, local gyrification index, and sulcal depth, were measured on the brain surface of the four brain lobes (frontal, parietal, temporal, and occipital lobes) (Supplementary Fig. 2g–l)^36–39^. Areas of the four lobes of WM surface were calculated as the summation of the areas formed by the triangular mesh^40^. To calculate local gyrification index and sulcal depth, convex hull surface of the 10 brain regions was first created^41^. Local gyrification index quantifies the amount of cortex buried within the sulcal folds, representing the extent of cortical folding. For each vertex on the surface, the local gyrification index is defined as the ratio between the area of a circular region of the vertex on the surface and the corresponding area on the convex hull for the vertex^42–44^. The sulcal depth was computed as the distance from each vertex on the brain surface to the nearest point on a convex hull for each hemisphere^45^. The surface area, local gyrification index and sulcal depth were calculated on the inner surface of the CGM (i.e., the gray and white junction)^36,37,46,47^.

### Statistics and reproducibility

Demographic data are presented as frequency and percent or median and quartile (25^th^, 75^th^), where appropriate. Data were explored for departures from normality using the Shapiro-Wilks test. Non-normally distributed parameters included gestational age (GA), maternal age, birth weight, birth head circumference, Apgar score, and maternal distress measures (i.e., stress, anxiety, depression). The fetal and maternal demographics were therefore compared between pre-pandemic and pandemic cohorts using non-parametric tests such as Wilcoxon-Mann-Whitney tests for continuous data and using Chi-square tests for categorical data.

Given that some mothers had repeated scans and thereby presented correlated data, we chose to use separate generalized estimating equations (GEEs) to examine fetal brain tissue volumes and cortical features in association with cohort status. GEE is a robust statistical method employed to study population-averaged patterns or trends over time for longitudinal data, allowing for multiple measurements per subject^48^. If an individual fetus was scanned at two time points in the fetal period, then both scans (if successful) were included for data analysis. Our modeling strategy was as follows. First (“Step 1”), we examined with separate models the associations between cohort status (pre-pandemic: 0 [referent]; pandemic: 1) and maternal distress measures (SSAI, STAI, PSS, and EPDS), adjusted for GA (weeks) at MRI and fetal sex^48,49^. In addition, the distress measures were further compared between pre-pandemic and pandemic cohorts in the low and high distress groups based on their corresponding threshold (SSAI: 40; STAI: 40; PSS: 15; EPDS: 10). Therefore, a total of 12 GEE models were implemented. Second (“Step 2”), separate GEE models were utilized to assess the associations between cohort status and fetal brain tissue volumes (i.e., brain tissue volumes for the six brain tissues) and cortical features (i.e., surface area, local gyrification index, and sulcal depth), controlled for GA at MRI (weeks), fetal sex, and each maternal distress measure (as a continuous variable) within each GEE model to determine whether cohort status was associated with fetal brain tissue volumes and cortical features. Specifically, 18 GEE models were adjusted for cohort status, GA at MRI, and fetal sex to determine the differences in each brain region between pre-pandemic and pandemic cohorts, with an additional 72 models that were further adjusted for each maternal distress measure, for a total of 90 GEE models implemented. Lastly (“Step 3”), the entire cohort was separated into high distress and low distress groups for each maternal distress measure based on published cut points (i.e., 40 for anxiety^24^, 15 for stress^25^, or 10 for depression)^29^ for those significant maternal distress measures for all subjects found in Step 1, and separate GEE models were conducted to investigate the association between cohort status and fetal brain tissue volumes and global cortical features (i.e., combined measures of the four lobes) in each group following adjustment for GA at MRI and fetal sex. Therefore, a total of 36 GEE models were implemented, where 24 models were for brain tissue volumes and 12 models were for cortical features.

Sub-analyses of other potential confounders were also implemented. First, we conducted GEE analyses for the associations between fetal brain volumes/brain cortical features and each maternal distress measure (treated as a continuous variable), adjusting for fetal sex and GA at MRI for all subjects (including both pre-pandemic and pandemic cohorts). A total of 72 GEE models were implemented. Second, we conducted the analysis of the GA-cohort status interaction for the brain cortical features, by further adjusting the GA-cohort status interaction in the GEE models in “Step 2” above, without adjusting for the maternal distress measures. A total of 12 GEE models were implemented. Third, two sensitivity analyses were conducted: (1) exclusion of scans performed below 28 weeks gestation and (2) exclusion of mothers greater than 40 years of age as potential outlier. A total of 180 GEE models were implemented (36 GEE models without adjustment for maternal distress measures and 144 GEE models with adjustment for maternal distress measures). Fourth, we evaluated potential differences in laterality by fitting the GEEs by the two hemispheres to investigate the effect of the cohort status on the fetal brain volumes/brain cortical features, adjusting for GA at MRI and fetal sex. A total of 36 GEE models were implemented. Lastly, the effect of parental education and employment on the fetal brain volumes/brain cortical features was examined. A total of 72 GEE models were implemented.

For demographic pre-pandemic vs. pandemic comparisons, statistical significance was assumed for *p* < 0.05, two-tailed. All subsequent *p* values were also adjusted for multiple testing using the false discovery rate method based on the number of outcomes (6 tissues or 4 lobes)^50^. All analyses performed in this study were conducted using MATLAB R2019a (The MathWorks, Inc., Natick, MA, USA)^48^.

### Reporting summary

Further information on research design is available in the Nature Research Reporting Summary linked to this article.

## Results

### Demographics

A diagram illustrating participant recruitment is shown in Supplementary Fig. 1. Seventy-two (21%) MRI scans (pre-pandemic: 62; pandemic: 10) with excessive fetal motion were excluded. The final data set consisted of 202 pregnant women (pre-pandemic: 137; pandemic: 65) between 16.7 to 39.1 gestational weeks, in which a total of 274 fetal brain MRI scans were acquired (Table 1). The distribution of fetal scans across time are presented in Supplementary Fig. 3. Seventy (26%) MRI scans (pre-pandemic: 34; pandemic: 36) failed brain surface reconstruction and therefore were not used for cortical folding calculations. Among the 202 study participants, 72 participants were scanned twice during pregnancy (45 pre-pandemic and 27 pandemic) while all other subjects were scanned once (92 pre-pandemic and 38 pandemic). The median GA at MRI was 30.2 weeks (range: 16.7 to 39.1) for the pre-pandemic group and was 30.8 weeks (range: 17 to 38.4) for the pandemic group. The median maternal age for the entire cohort was 34.1 years old (range: 17 to 51). The median GA at birth was 39.6 weeks (range: 31.0 to 41.9), and the median birth weight was 3.36 kg (range: 1.02 to 4.70). 100 (52%) of fetuses were male, and 91 (48%) were female. No significant differences of the parental education and employment distributions were observed between pre-pandemic and pandemic cohorts (Supplementary Table 1).

**Table 1:** Demographics of 202 pregnant women who underwent 274 prenatal MRI studies.

*N* [%] or Median [IQR]	All Subjects	Pre-pandemic	Pandemic	*p*
Number of subjects	202	137	65	
Female fetus	91 [48]	58 [45]	33 [54]	0.22
Male fetus	100 [52]	72 [55]	28 [46]	
With 1 scan	130 [64]	92 [67]	38 [58]	0.23
With 2 scans	72 [36]	45 [33]	27 [42]	
Number of MR scans	274	182	92	
Time point 1	147 [54]	101 [55]	46 [50]	0.39
Time point 2	127 [46]	81 [45]	46 [50]	
GA at MRI	30.4	30.2	30.8	0.15
	[26.1, 35.3]	[27.0, 35.9]	[25.3, 34.1]	
Maternal age (years)^*^	34.1	34.0	34.6	0.63
	[31.0, 36.9]	[31.0, 37.8]	[30.5, 36.1]	
Maternal parity (primiparous/multiparous)^†^	109/80	73/55	36/25	0.80
GA at birth (weeks)^‡^	39.6	39.7	39.4	0.51
	[38.7, 40.4]	[38.4, 40.6]	[38.9, 40.1]	
Birth weight (kg)^§^	3355	3382	3285	0.91
	[3078, 3677]	[3079, 3670]	[3064, 3690]	
Birth head circumference (cm)^#^	34.3	34.0	34.5	0.19
	[33.5, 35.5]	[33.0, 35.0]	[33.7, 35.6]	
Apgar score at 1 min^††^	8	8	8	0.66
	[8, 9]	[8, 9]	[8, 9]	
Apgar score at 5 min^‡‡^	9	9	9	0.32
	[9, 9]	[9, 9]	[9, 9]	
Delivery Mode^§§^				0.10
Vaginal	101 [68]	59 [62]	42 [78]	
Elective C-section	26 [17]	18 [19]	8 [15]	
Emergency C-section	22 [15]	18 [19]	4 [7]	
Race/Ethnicity^##^				0.07
White	100 [57]	68 [56]	32 [58]	
Black	33 [19]	25 [21]	8 [15]	
Hispanic or Latino	20 [11]	12 [10]	8 [15]	
Asian or Pacific Islander	10 [6]	4 [3]	6 [11]	
Others	13 [7]	12 [10]	1 [2]	

*N* = 274 MR scans for volumetric data, and *N* = 204 MR scans for cortical features. *GA* Gestational age. *IQR* Interquartile range. *: Based on 200 (99%) subjects. †: Based on 189 (94%) subjects. ‡: Based on 165 (82%) subjects. §: Based on 156 (77%) subjects. #: Based on 108 (53%) subjects. ††: Based on 146 (72%) subjects. ‡‡: Based on 146 (72%) subjects. §§: Based on 149 (74%) subjects. ##: Based on 176 (87%) subjects.

### Pandemic related differences in fetal brain tissue volumes and morphometry

**Fig. 1:**
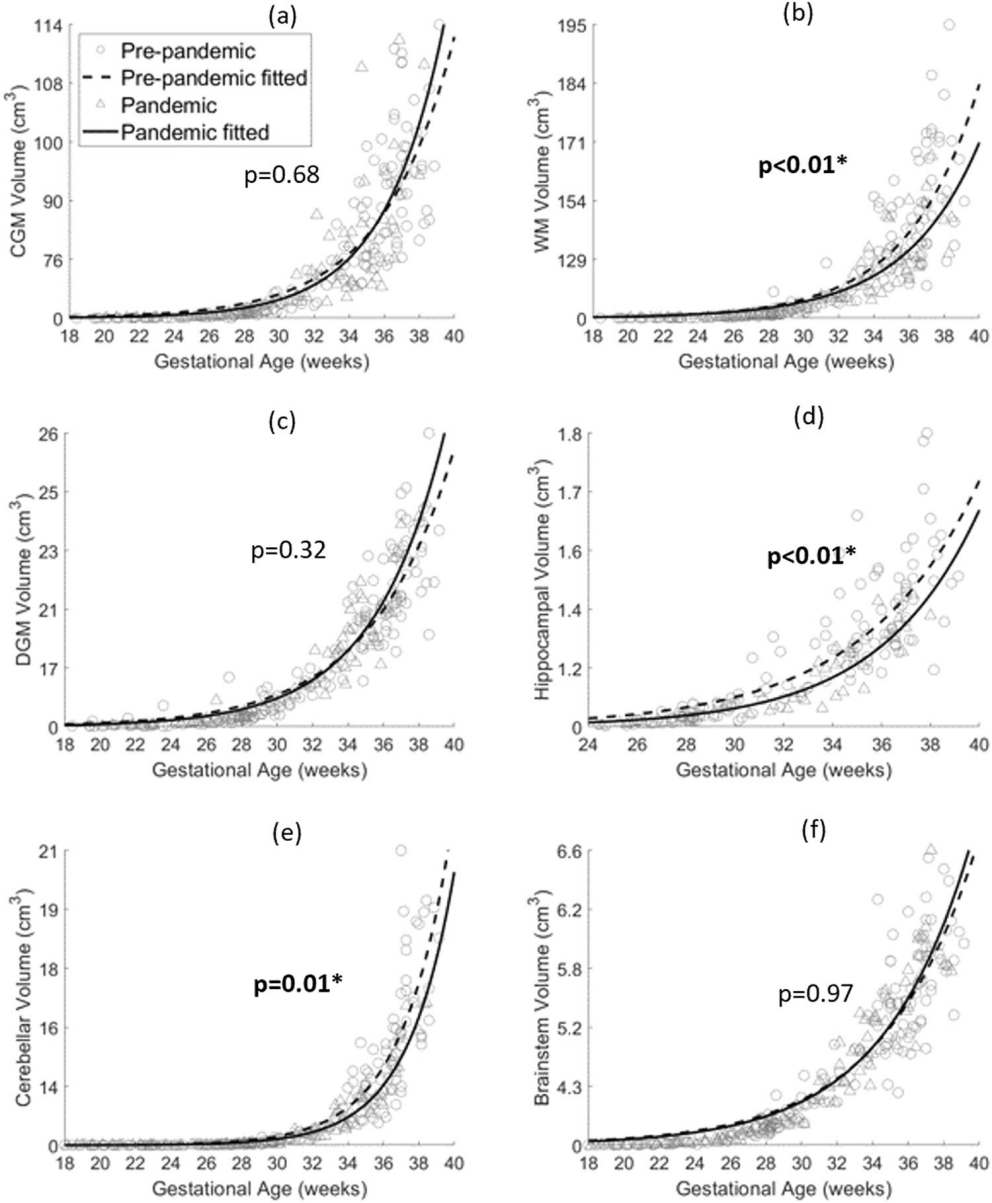
Scatter plots with exponential fits of brain tissue volumes vs. gestational age. **a** Cortical gray matter (CGM); **b** White matter (WM); **c** Cerebellum; **d** Deep gray matter (DGM); **e** Brainstem; **f** Hippocampus. The *p*-values are the main effects of pandemic vs. pre-pandemic on brain tissue volumes while controlling for gestational age at MRI and fetal sex. Bold *p*: *p* < 0.05. *: *q* < 0.05. Sample size *N* = 274.

**Fig. 2:**
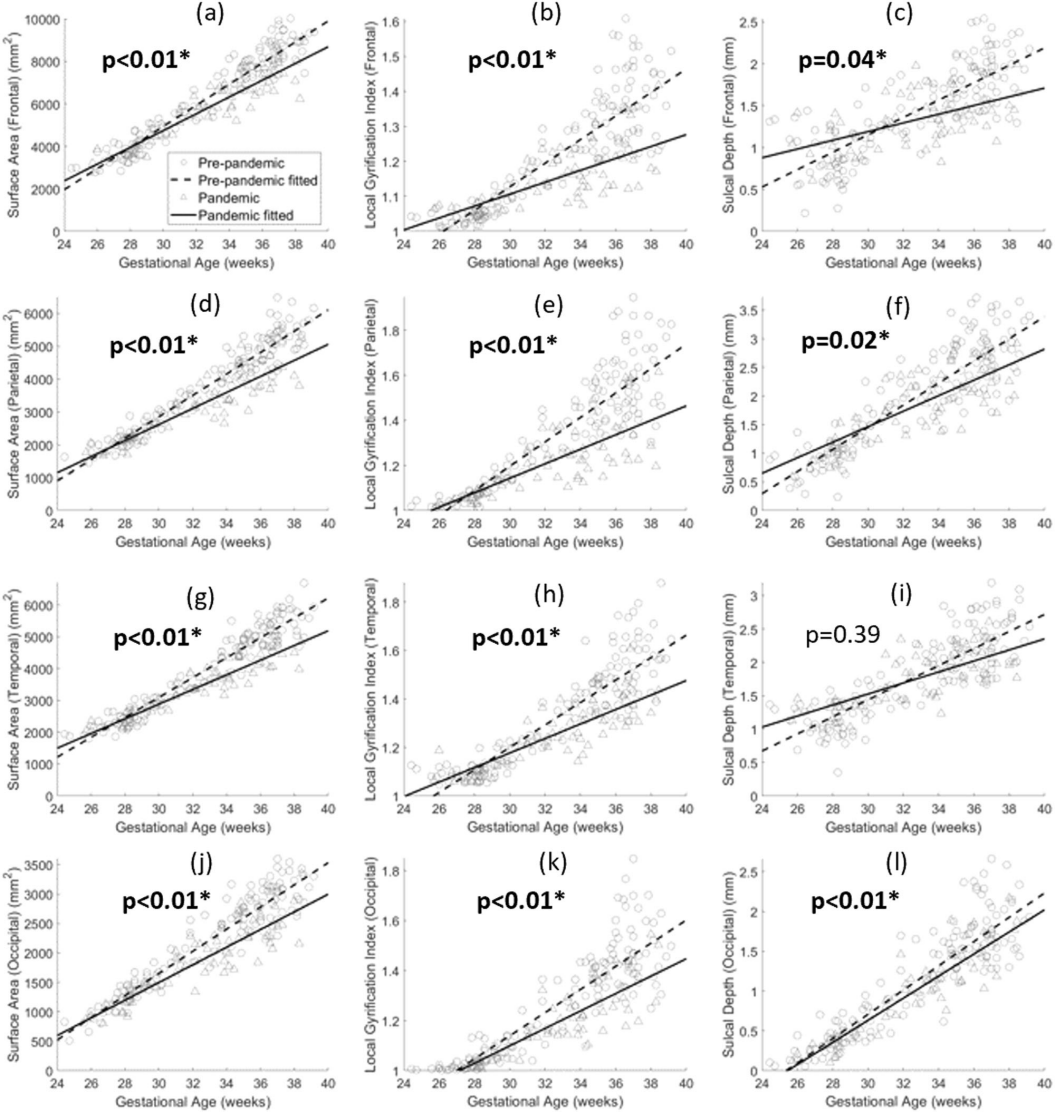
Scatter plots with linear fits of brain lobe surface area/LGI/sulcal depth vs. gestational age. **a**–**c**: Frontal lobe; **d**–**f** Parietal lobe; **g**–**i**: Temporal lobe; **j**–**l**: Occipital lobe. The *p*-values are the main effects of pandemic vs. pre-pandemic on brain cortical features while controlling for gestational age at MRI and fetal sex. Bold *p*: *p* < 0.05. *: *q* < 0.05. Sample size *N* = 204.

Smaller fetal brain WM, hippocampal, and cerebellar volumes were observed in the pandemic cohort when controlling for GA at MRI (weeks) and fetal sex in GEE models (least squares means: 93.3 vs. 99.1 cm^3^, *p* < 0.01 in WM, 8.2 vs. 8.7 cm^3^, *p* = 0.01 in cerebellum, and 1.0 vs. 1.1 cm^3^, *p* < 0.01 in hippocampus) (Fig. 1). Regional cortical features were calculated for the four lobes: frontal, parietal, temporal, and occipital lobes. Lower surface area and local gyrification indices were found in the pandemic cohort for all four lobes, compared to the pre-pandemic cohort, while sulcal depth was lower in the pandemic cohort for frontal, parietal, and occipital lobes when controlling for GA at MRI (weeks) and fetal sex in GEE models (Fig. 2).

### Associations with gestational age

The results of the effect of GA-cohort status interaction on the brain cortical features are shown in Supplementary Table 2. For the brain area and local gyrification index, the GA-cohort status interactions were significant for all four brain lobes. For the sulcal depth, the GA-cohort status interactions were significant for the frontal, parietal, and temporal lobes. Given these and the known influence of GA on brain development, GA at the time of MR was included in all models.

### Differences between the two hemispheres

We fitted the GEEs for each cerebral and cerebellar hemisphere to investigate the association of COVID-19 on the fetal brain laterality, adjusting for GA at MRI and fetal sex. We found similar differences in fetal brain volume and morphometry between left and right hemispheres when comparing pandemic to pre-pandemic cohorts (Supplementary Table 3). Specifically, we observed significant differences of white matter and hippocampal volumes for both hemispheres between pre-pandemic and pandemic cohorts, while cerebellar volume was associated with pandemic status for the left hemisphere only. Surface area and gyrification indices were uniformly decreased for left and right hemispheres in the pandemic cohort compared to the pre-pandemic cohort, while only sulcal depth of the left frontal parietal and occipital lobes was significantly decreased in the pandemic cohort compared to the pre-pandemic cohort, when accounting for multiple comparisons.

### Associations with fetal sex

We observed higher CGM volume in male fetuses when compared to female fetuses (*p* < 0.05), even when adjusting for GA and maternal distress measures. In addition, male fetuses had larger surface areas on frontal, parietal, and occipital lobes than female fetuses when adjusting for GA at MRI. However, fetal sex had no effect on fetal brain volumes/brain cortical features after adjusting for multiple comparisons for any of these associations.

### Associations with parental education and employment

We further examined the effect of parental education and employment on the fetal brain volumes and cortical features (Supplementary Table 4). The results showed that the maternal education was positively associated with cerebellar volume (Supplementary Table 4). The paternal education was positively associated with WM and brainstem volumes; however, it was negatively associated with the local gyrification index in the parietal and occipital lobes and associated with the sulcal depth in the occipital lobe (Supplementary Table 4). Paternal employment was negatively associated with local gyrification index in the frontal, parietal, and temporal lobes (Supplementary Table 4).

### Maternal psychological distress

Among 173 pregnant women with available maternal distress measures, 34 (27.6%) women in the pre-pandemic cohort and 26 (52.0%) women in the pandemic cohort were considered to have elevated maternal psychological distress (the high distress group) if at least one of the four distress measures were greater than the predefined threshold (SSAI > 40; STAI > 40; PSS > 15; EPDS > 10). Overall, mean scores of maternal stress and depression were significantly higher in the pandemic cohorts (Supplementary Table 5). Using the predefined thresholds for elevated scores of maternal mental distress, we further compared mean scores among low and high distress groups (Supplementary Table 5). No significant differences were observed between pre-pandemic and pandemic cohorts in the high distress group (Supplementary Table 5); however, mean anxiety and depression scores were significantly higher (though below the predefined threshold) in the pandemic cohort compared to the pre-pandemic cohort in the low distress group (Supplementary Table 5).

For all subjects combined, elevated maternal anxiety (SSAI and STAI) and stress (PSS) were associated with smaller hippocampal and cerebellar volumes, and higher STAI was associated with lower WM volume (Supplementary Table 6). In addition, elevated maternal anxiety (SSAI and STAI) and depression (EPDS) were associated with higher sulcal depth (Supplementary Table 6).

Adjusting for maternal distress measure, the WM, hippocampal, and cerebellar volumes remained smaller in the pandemic cohort, compared to the pre-pandemic cohort (Table 2). Furthermore, comparisons between low and high maternal distress for each cohort revealed lower WM, cerebellar, and hippocampal volumes were observed in the pandemic cohort with low maternal stress group (i.e., low PSS), (91.1 vs. 98.5 cm^3^, *p* < 0.01 in WM, 8.2 vs. 8.8 cm^3^, *p* = 0.04 in cerebellum, and 1.06 vs. 1.16 cm^3^, *p* < 0.01 in hippocampus) (Table 3). Similarly, in the pandemic cohort with low maternal depression (i.e., low EPDS), lower WM and hippocampal volumes were observed (95.0 vs. 100.0 cm^3^, *p* = 0.02 in WM and 1.06 vs. 1.14 cm^3^, *p* < 0.01 in hippocampus) (Table 3).

**Table 2:** The results of the generalized estimating equations (GEEs) for the associations between fetal brain volumes/brain cortical features and cohort status (0: pre-pandemic; 1: pandemic), adjusting for fetal sex, gestational age at MRI (weeks) and each maternal distress measure.

	Brain volumes (mm^3^)							
	SSAI		STAI		PSS		EPDS	
	*β*	*p*	*β*	*p*	*β*	*p*	*β*	*p*
CGM	660	0.64	734	0.60	817	0.55	349	0.81
WM	−5154	0.01*	−4903	0.01*	−5096	0.01*	−5479	0.01*
DGM	−92	0.71	−63	0.80	3.8	0.99	−143	0.56
Hippocampus	−76	<0.01*	−74	<0.01*	−67	<0.01*	−72	<0.01*
Cerebellum	−468	0.02*	−442	**0.03**	−412	0.05	−484	0.02*
Brainstem	1.9	0.97	8.1	0.86	−9.3	0.85	−6.3	0.89
	Lobe Surface Area (mm^2^)							
	SSAI		STAI		PSS		EPDS	
	*β*	*p*	*β*	*p*	*β*	*p*	*β*	*p*
Frontal	−460	<0.01*	−455	<0.01*	−478	<0.01*	−476	<0.01*
Parietal	−463	<0.01*	−460	<0.01*	−472	<0.01*	−474	<0.01*
Temporal	−428	<0.01*	−429	<0.01*	−446	<0.01*	−441	<0.01*
Occipital	−258	<0.01*	−254	<0.01*	−274	<0.01*	−268	<0.01*
	Local Gyrification Index (×10^−3^)							
	SSAI		STAI		PSS		EPDS	
	*β*	*p*	*β*	*p*	*β*	*p*	*β*	*p*
Frontal	−69	<0.01*	−68	<0.01*	−69	<0.01*	−68	<0.01*
Parietal	−117	<0.01*	−116	<0.01*	−117	<0.01*	−117	<0.01*
Temporal	−76	<0.01*	−77	<0.01*	−83	<0.01*	−80	<0.01*
Occipital	−73	<0.01*	−73	<0.01*	−76	<0.01*	−74	<0.01*
	Sulcal Depth (×10^−3^ mm)							
	SSAI		STAI		PSS		EPDS	
	*β*	*p*	*β*	*p*	*β*	*p*	*β*	*p*
Frontal	−113	**0.04**	−119	0.04*	−123	0.04*	−123	0.03*
Parietal	−157	0.02*	−167	0.02*	−181	0.01*	−181	0.01*
Temporal	−73	0.15	−80	0.13	−96	0.08	−87	0.09
Occipital	−113	0.01*	−115	0.01*	−123	0.01*	−125	0.01*

The *β* and *p* represent the coefficient and its significance of the cohort status in each GEE. *SSAI* Spielberger State Anxiety Inventory. *STAI* Spielberger Trait Anxiety Inventory. *PSS* Perceived Stress Scale. *EPDS* Edinburgh Postnatal Depression Scale. *CGM* Cortical gray matter. *WM* White matter. *DGM* Deep gray matter. Bold *p*: *p* < 0.05. *: *q* < 0.05.

**Table 3:** Comparisons of brain tissue volumes (least squares mean ± standard error) between pre-pandemic and pandemic cohorts from the generalized estimating equations for the associations between fetal brain volumes (cm^3^) and cohort status (0: pre-pandemic; 1: pandemic), adjusting for gestational age at MRI (weeks) and fetal sex.

	Pre-pandemic	Pandemic	*p*
Low PSS			
CGM	59.6 ± 4.0	58.3 ± 4.2	0.41
WM	98.5 ± 6.6	91.1 ± 7.0	<0.01*
DGM	15.4 ± 1.0	15.4 ± 1.0	0.99
Hippocampus	1.16 ± 0.08	1.06 ± 0.08	<0.01*
Cerebellum	8.76 ± 0.92	8.23 ± 0.95	**0.04**
Brainstem	3.94 ± 0.13	3.91 ± 0.14	0.61
High PSS			
CGM	62.5 ± 6.7	64.7±7.0	0.30
WM	99.9 ± 9.7	98.2 ± 10.3	0.60
DGM	15.6 ± 1.1	15.9±1.1	0.25
Hippocampus	1.05 ± 0.07	1.01 ± 0.08	0.22
Cerebellum	8.87 ± 1.55	8.38 ± 1.59	0.13
Brainstem	4.05 ± 0.20	4.06 ± 0.21	0.86
Low EPDS			
CGM	60.5 ± 3.6	61.0 ± 3.9	0.74
WM	100.0 ± 5.9	95.0 ± 6.3	**0.02**
DGM	15.6 ± 1.0	15.7 ± 1.0	0.63
Hippocampus	1.14 ± 0.06	1.06 ± 0.06	<0.01*
Cerebellum	8.88 ± 0.83	8.44 ± 0.87	0.06
Brainstem	3.99 ± 0.11	4.01 ± 0.12	0.65
High EPDS			
CGM	58.7 ± 13.7	55.6 ± 14.0	0.31
WM	89.9 ± 20.9	84.2 ± 21.6	0.29
DGM	14.7 ± 3.2	14.3 ± 3.2	0.51
Hippocampus	0.94 ± 0.11	0.90 ± 0.12	0.34
Cerebellum	7.59 ± 2.50	6.91 ± 2.54	0.16
Brainstem	3.78 ± 0.35	3.60 ± 0.37	0.12

*CGM* Cortical gray matter. *WM* White matter. *DGM* Deep gray matter. *PSS* Perceived Stress Scale. *EPDS* Edinburgh Postnatal Depression Scale. Bold *p*: *p* < 0.05. *: *q* < 0.05.

After further adjusting for each maternal distress measure on morphometric features, surface area and local gyrification indices remained lower in the pandemic cohort for all four lobes, and sulcal depth also remained lower in the pandemic cohort for the frontal, parietal, and occipital lobes (Table 2). Global surface area and local gyrification indices were lower in both the low and high stress pandemic cohorts, compared to the pre-pandemic cohorts (145.4 vs. 162.0 cm^2^, *p* < 0.01 for surface area in low PSS group, 143.3 vs. 160.6 cm^2^, *p* < 0.01 for surface area in high PSS group, 1.20 vs. 1.28, *p* < 0.01 for local gyrification index in low PSS group, and 1.20 vs. 1.30, *p* < 0.01 for local gyrification index in high PSS group) (Table 4) as well as both the low and high depression pandemic cohorts, compared to the pre-pandemic cohorts (146.2 vs. 161.1 cm^2^, *p* < 0.01 for surface area in low EPDS group, 134.4 vs. 154.5 cm^2^, *p* < 0.01 for surface area in high EPDS group, 1.20 vs. 1.28, *p* < 0.01 for local gyrification index in low EPDS group, and 1.16 vs. 1.27, *p* = 0.02 for local gyrification index in high EPDS group) (Table 4). However, sulcal depth was significantly decreased in the high stress pandemic cohort only, compared to the pre-pandemic cohort (1.50 vs. 1.66 mm, *p* = 0.02) (Table 4), and the low depression pandemic cohort, compared to the pre-pandemic cohort (1.51 vs. 1.61 mm, *p* = 0.045) (Table 4).

**Table 4:** Comparisons of brain lobe surface area (cm^2^)/LGI/sulcal depth (mm) (least squares mean ± standard error) between pre-pandemic and pandemic cohorts from the generalized estimating equations for the associations between cohort status (0: pre-pandemic; 1: pandemic) and brain lobe surface area/LGI/sulcal depth of combined four brain lobes, adjusting for gestational age at MRI (weeks) and fetal sex.

	Pre-pandemic	Pandemic	*p*
Low PSS			
Surface Area	162.0 ± 10.2	145.4 ± 10.9	<0.01*
Local Gyrification Index	1.28 ± 0.06	1.20 ± 0.06	<0.01*
Sulcal Depth	1.60 ± 0.23	1.49 ± 0.24	0.07
High PSS			
Surface Area	160.6 ± 16.8	143.3 ± 17.1	<0.01*
Local Gyrification Index	1.30±0.12	1.20 ± 0.12	<0.01*
Sulcal Depth	1.66 ± 0.38	1.50 ± 0.38	0.02*
Low EPDS			
Surface Area	161.1 ± 9.1	146.2 ± 9.5	<0.01*
Local Gyrification Index	1.28 ± 0.06	1.20 ± 0.06	<0.01*
Sulcal Depth	1.61 ± 0.20	1.51 ± 0.21	0.045*
High EPDS			
Surface Area	154.5 ± 32.3	134.4 ± 32.9	<0.01*
Local Gyrification Index	1.27 ± 0.24	1.16 ± 0.25	0.02*
Sulcal Depth	1.58 ± 0.70	1.34 ± 0.72	0.12

*PSS* Perceived Stress Scale. *EPDS* Edinburgh Postnatal Depression Scale. Bold *p*: *p* < 0.05. *: *q* < 0.05.

### Sensitivity analysis

Excluding scans acquired before 28 weeks gestation, GEEs independent of maternal distress measures were unchanged. After adjusting for each maternal distress measure, the findings also remained similar (compared to Table 2) with the exception that the WM volume was not associated with the cohort status (Supplementary Table 7). Excluding mothers greater than 40 years of ages, GEEs were independent of maternal distress measures were unchanged. Adjusting for each maternal distress measure, the findings also remained similar with the exception that the sulcal depth in the frontal lobe was not associated with the cohort status (Supplementary Table 8).

## Discussion

### Summary of findings

This study utilized advanced in vivo fetal 3D volumetric MRI to investigate the impact of the COVID-19 pandemic status on *in utero* fetal brain development during the latter half of gestation. The COVID-19 pandemic has had widespread impact on societal health and well-being extending well beyond the morbidity and mortality of acquired infections^51–57^. It is increasingly recognized that alterations in the intrauterine environment, including fetal exposure to maternal psychological distress, can adversely influence early fetal brain development and subsequent neurobehavioral health in offspring^3,58–60^. In this work, we found elevated levels of maternal stress and depression in pregnant women during the pandemic, similar to previously published work. We further demonstrated decreased fetal WM, hippocampal, and cerebellar volumes during the pandemic compared to a cohort of pre-pandemic pregnant women and fetuses, along with decreased brain surface area and gyrification in the fetuses of pregnant women studied during the pandemic. We also report a negative association between maternal stress and anxiety with fetal hippocampal and cerebellar volumes overall, as well as a positive association between sulcal depth of the fetal temporal lobe and maternal depression and anxiety. Adjusting for maternal distress measures, we show a persistent association between maternal anxiety, stress and depression with decreased WM, hippocampal and cerebellar volumes between pandemic and pre-pandemic cohorts, as well as negative associations between maternal mental distress and global measures of fetal surface area, gyrification and sulcal depth. Our data suggest the cumulative and downstream effects of the COVID-19 pandemic increase prenatal maternal psychological distress may further contribute to the altered development of structures in key regions of the fetal brain.

It is interesting to note, however, we did not find increased rates of maternal anxiety in our pandemic cohort, considering recent meta-analyses have reported increased anxiety among pregnant women during the COVID-19 pandemic^61,62^. However, as recent literature highlights, there is substantial heterogeneity in published results of anxiety in pregnant women during the pandemic^61,62^. This may be due to the type of tool used and assessment style^62^, as well as timing of assessment and geographic variability^61^. In these and other studies, it has been proposed that anxiety or panic may be widespread in certain regions with fast growing COVID cases or lack of medical support^63–65^, along with data revealing a higher prevalence later in the pandemic^61^. Our participants were recruited in the region of Washington D.C. between 2020 and 2021, where COVID cases were relatively well-controlled compared to other major cities in the U.S^66–68^. and relied on maternal response to the STAI questionnaires to identify elevated anxiety, which may account for these differences.

### Regional brain volumes and mental health

In this study, we found that cerebral WM, hippocampal, and cerebellar volumes were lower in the pandemic cohort, compared to the pre-pandemic cohort, and were negatively associated with anxiety, stress, and depression scores. Previous studies highlight several changes in brain volumes for offspring exposed to prenatal depression throughout childhood. In infancy, subcortical GM is increased, and midbrain volumes are decreased for children born to mothers with major depressive disorders in pregnancy^7^. By age 10, GM is decreased in a group of nearly 4000 children^69^, though this finding may be driven more by postnatal depression exposure than prenatal symptoms^69^. Interestingly, this study also found that children of mothers with high perinatal symptoms of prenatal depression had 3.4% less total WM volume compared to children of mothers with no/low depressive symptoms^69^, similar to our findings in the fetus. Changes in WM development are associated with behavioral problems in infancy^70^, social-emotional processing, language, and memory problems by school age^71^, as numerous psychiatric conditions, including generalized and social anxiety disorders, depression, post-traumatic stress, and autism spectrum disorders^72,73^. Conversely, there are multiple reports of increased amygdalar volume in the neonatal period^74^ that persist through 4.5 years of age^75^ for female children born to mothers with elevated prenatal depression. This corresponds with a smaller study that found smaller amygdalar volumes in boys^76^. The mechanisms behind these differences remain unclear; however, maternal cortisol, especially early in gestation, also has been associated with increased amygdalar volume in girls at age 7, along with increased affective problems^77^. It remains unclear if this change persists throughout childhood, as the study by Zou et al. found no differences in volumes of the amygdala or hippocampus at age 10^69^. Much less is known about fetal hippocampal and cerebellar volumes and later neuropsychiatric morbidity. In adults, decreased hippocampal volume, however, is associated with psychiatric disorders, including post-traumatic stress disorder^77^ and major depressive disorder (MDD)^78–81^, and cerebellar maldevelopment is associated with neurobehavioral and psychiatric morbidity in older children^82^.

While WM, hippocampal, and cerebellar volumes were decreased in our pandemic cohort, compared to the pre-pandemic cohort, it is important to note that when stratified into high- and low-scores, fetuses of pregnant women in the low stress group had lower volumes across all three brain regions in the pandemic cohort compared to the pre-pandemic cohort. These data, along with previously published reports that reveal distinct and at times inconsistent differences in brain volumes across childhood^7,69,74–77^, suggest that there are likely multiple factors that influence fetal brain volume across the lifespan. These factors may include unmeasured factors specific to the COVID-19 pandemic, including social isolation, financial insecurity, and nutritional changes that remain unaccounted. The variability in these data also suggest that differences in brain structure across time may reflect periods of both vulnerability and plasticity and may allow for multiple windows of interventions for both mother and child.

### Cortical maturation and sulcal depth

We further report global reductions in cerebral surface area and gyrification indices in the pandemic cohort, adjusting for maternal stress, anxiety, and depression scores, and only note relative sparing of temporal lobal sulcal depth in the setting of maternal psychological distress. It is interesting to note that the effect of maternal psychological distress for all subjects across both epochs, however, had a positive association with the sulcal depth of the temporal lobe, though this association did not remain after adjusting for multiple comparisons. Numerous studies have identified aberrations in cortical structure in children, adolescents, and adults with depression, anxiety, stress and other neuropsychiatric conditions, including differences in cortical thickness, surface area and gyrification^83–95^. In children imaged at 4 and 6 years of age, female offspring of mothers with elevated prenatal depressive symptoms also had decreased surface areas of the dorsolateral prefrontal cortex, anterior superior temporal gyrus, and right superior parietal lobe, while male offspring had increased surface areas in these regions, as well as increased surface areas of the right lateral orbitofrontal cortex, anterior inferior temporal gyrus, left fusiform, and paracentral cortex^96^. In a study of slightly older children, prenatal maternal depressive symptoms were associated with increased surface area of the left caudal mid-frontal area at age 8, as well as thinning of the left superior frontal cortex^5^. Global cortical thinning, especially the frontal lobes, was also reported in children of both sexes exposed to prenatal depression at age 7^97^, the left inferior frontal cortex in female offspring at age 4.5 years^74^, and right frontal and temporal regions in a group of children between 2.6 and 5.1 years of age^98^. Cortical thinning also mediated child externalizing behaviors in children exposed to prenatal maternal depression at age 7^97^, and correlated with adolescent depressive symptoms in offspring at age 12^99^. It is interesting to note that we did not observe sex-related differences in brain development during the fetal period when accounting for multiple comparisons. However, previous work on fetal brain volumes similarly did not detect significant sex differences during gestation^100^.

### Prenatal stressors and neurodevelopment

It is increasingly recognized that intrauterine exposure to any numbers of stressors may adversely impact fetal neurodevelopment^60,101^. Until recently, a substantial challenge has been to separate prenatal from postnatal exposures that could adversely affect offspring neurodevelopment; advances in quantitative fetal MRI, however, allow for the real-time evaluation of prenatal stress on fetal brain structure and function^10–12,14^. Maternal psychological distress, including stress, anxiety, and depressive symptoms may disrupt critical neuroendocrine functions along with the development of the hypothalamic-pituitary-adrenal axis and autonomic nervous system^101,102^. Similarly, there is emerging evidence of the bidirectional interplay of maternal nutrition and stress in pregnancy on fetal brain development^103^, and increased inflammation in prenatal stress and depression^65,92,102,104,105^. The neurologic underpinnings of psycho-behavioral disorders remain complex and challenging to elucidate fully. Conventional neuroimaging can aid the identification of neurologic diseases that may present with psychiatric symptoms^106^, while advanced quantitative and functional MRI techniques reveal subtle but important alterations in brain morphometry and network dysfunction that contribute to psycho-behavioral disorders^107^. Noteworthy, early cortical folding patterns underpin emerging functional and structural connectivity in the developing brain^84,108,109^. During the fetal period, the cortex undergoes rapid and substantial changes in morphometry with sequential windows of vulnerability to individual stressors; studies such as ours now allow for the real-time evaluation of early stressors on emerging brain development and provide a better mechanistic understanding of the intrauterine programming effects that predispose offspring to neuropsychiatric disease later in life. However, the evolution of these early findings across the developmental lifespan remains largely unknown. Recent studies, however, suggest important associations between exposure to prenatal maternal anxiety or depression, altered cortical morphometry and adverse neuropsychiatric behaviors into early adolescence^97,99^.

We report that parental education and employment status were also associated with fetal brain development. The relationship between parental education and employment status with neurodevelopment in infants and older children has been previously described^110–115^. Our previous study established similar associations between parental socioeconomic status (education, occupation, and socioeconomic status scores) and altered in vivo fetal brain regional volumes and cortical folding in a healthy fetal cohort before the pandemic^14^. These associations further suggest that a variety of early life psychosocial stressors may contribute to childhood brain development^110,116,117^ and also highlight unique opportunities for intervention that may optimize outcomes^118^.

### Limitations

Our study limitations deserve mention. First, the COVID-19 pandemic may result in any number of lifestyle changes that can influence maternal health and fetal development. In this study, we examined whether maternal distress, both during and before the COVID-19 pandemic, was associated with fetal neurodevelopment given the known association of prenatal stress, anxiety, and depression on offspring outcomes^10–13,119^. Similar to previous studies, we found a significant association with adverse prenatal exposures and disrupted fetal brain development, namely, reductions in regional fetal brain volumes, cortical surface area, and gyrification. Nonetheless, it is important to note that our findings may not be solely related to maternal mental distress. Indeed, though we identified decreased global surface area, gyrification and sulcal depth in the pandemic groupings of high stress and high depression, these associations did not remain after multiple comparisons. These findings suggest the presence of other pandemic-specific stressors that contribute to early brain volumes. Similarly, while parental education and employment were similar between cohorts, these factors have been independently associated with offspring neurodevelopment. Detailed examination of these and other influences on maternal-fetal health is warranted, including comorbid stress and depression, maternal nutrition, financial security, familial psychopathology, and genetic factors, to better understand these associations. It is also important to note that women recruited in this study were from the Washington, DC metropolitan area and predominantly self-identified as white and black; the associations observed in this study should be explored in other geographic and racial populations given the known regional heterogeneity in the experience of the COVID-19 pandemic, as well as the known racial and ethnic differences in adult brain structure^120,121^. While this study included women without confirmed COVID-19 exposures, it is possible women may have had unknown exposures or subclinical infections. The long-term impact of our findings, as well as known COVID-19 exposures on fetal brain development, is unknown and warrants further study. Furthermore, it should be noted that multiple comparisons correction was performed within each set of statistical tests, but not across all comparisons made. This approach was used to maximize the detection of critical factors in clinical data analysis for this exploratory study, while balancing the risk of false discovery^122^. Finally, the long-term neurodevelopmental consequences of these in vivo fetal brain alterations as measured by prenatal quantitative MRI are unknown and currently under investigation.

## Conclusions

Instances of both man-made and natural disasters have exposed the impact of prenatal stress and neurobehavioral effects on surviving offspring^3,56,76,123–126^. Given the breadth, depth, and duration of the current COVID-19 pandemic that has persisted across the globe, we are in a unique point in history to discover the short- and long-term significance of prenatal stress on early neurodevelopment, with the opportunity to implement and evaluate novel and timely interventions. While the COVID-19 pandemic may be a unique stressor given the number of people affected, lessons learned from this pandemic may be applicable to early-life stressors across multiple domains that may be applied to high-risk conditions independent of and subsequent to this pandemic. Understanding how contemporary stressors may influence fetal brain development during pregnancy has major implications for both answering basic scientific questions and informing public policy initiatives. Indeed, early studies now show that infant development of children born during the pandemic may be adversely affected, particularly when compared to pre-pandemic controls^127^. As we continue to elucidate the mechanisms underpinning these differences, concurrent efforts should emphasize the implementation of intervention programs for both maternal-infant dyads. Furthermore, monitoring the COVID generation of infants for long-term cognitive and health outcomes after birth is warranted and currently underway. Moreover, continued research efforts may inform preventive strategies for future pregnant women facing a multitude of psychosocial stressors beyond the current COVID-19 pandemic.

## Supplementary information

Supplementary Information

Supplementary Data 1

Supplementary Data 2

Description of Additional Supplementary Files

Reporting Summary

## Data Availability

All source data for figures in the main manuscript are contained in Supplementary Data 1 and Supplementary Data 2. Additional datasets are available upon direct request to corresponding authors. Requests to access additional datasets will undergo internal review and released pending necessary data or material transfer agreements.

## Code availability

The custom Matlab codes for analyzing the cortical features are available on Zenodo.org (10.5281/zenodo.6413064).
