# Supplementary material for: Maternal psychological distress during the COVID-19 pandemic and structural changes of the human fetal brain
Source: Commun Med (Lond). 2022 May 26;2:47. doi: 10.1038/s43856-022-00111-w (PMC9135751; doi:10.1038/s43856-022-00111-w)
Supplement: Supplementary file 1 — Supplementary Information [file 43856_2022_111_MOESM1_ESM.pdf]

Supplementary Table 1. Parental education and employment.

	All Subjects		Pre-pandemic		Pandemic		
	N	%	N	%	N	%	p
Maternal Education							
Partial High School	2	1	1	1	1	2	
High School Graduate	9	4	8	6	1	2	
Partial College (at least 1 year)	20	10	16	12	4	6	0.40
College or University Graduate	57	28	41	30	16	25	
Graduate professional Training	109	54	66	48	43	66	
Unknown	5	2	5	4	0	0	
Paternal Education							
Partial High School	5	2	4	3	1	2	
High School Graduate	15	7	12	9	3	5	
Partial College (at least 1 year)	15	7	12	9	3	5	0.70
College or University Graduate	54	27	33	24	21	32	
Graduate professional Training	93	46	63	46	30	46	
Unknown	20	10	13	9	7	11	
Maternal Employment							
Homemaker, Unemployed	23	11	15	11	8	12	
Unskilled laborer, Machine operative, Semiskilled operator	4	2	3	2	1	2	
Skilled craftsman, Clerical, Sales	6	3	6	4	0	0	0.27
Medium business, Minor professional, Technical	21	10	13	9	8	12	
Major business, Professional	141	70	94	69	47	72	
Unknown	7	3	6	4	1	2	
Paternal Employment							
Homemaker, Unemployed	10	5	7	5	3	5	
Unskilled laborer, Machine operative, Semiskilled operator	10	5	7	5	3	5	
Skilled craftsman, Clerical, Sales	8	4	8	6	0	0	0.66
Medium business, Minor professional, Technical	15	7	9	7	6	9	
Major business, Professional	140	69	94	69	46	71	
Unknown	19	9	12	9	7	11	

Note: P-values were calculated based on the Chi-square test between pre-pandemic and pandemic cohorts for known education and employment status.

Supplementary Table 2. The results of the generalized estimating equations (GEEs) for the associations between brain cortical features and cohort status (0: pre-pandemic; 1: pandemic), adjusting for gestational age (GA) at MRI (weeks), fetal sex, and GA-cohort interaction.

	Lobe Surface Area (cm^2)					
	GA		Cohort Status		GA-cohort Interaction	
	β	p	β	p	β	p
Frontal	4.99	**<0.01***	30.2	**<0.01***	-1.06	**<0.01***
Parietal	3.27	**<0.01***	23.3	**<0.01***	-0.85	**<0.01***
Temporal	3.14	**<0.01***	23.7	**<0.01***	-0.86	**<0.01***
Occipital	1.89	**<0.01***	10.5	**<0.01***	-0.40	**<0.01***
	Local Gyrification Index ($\times 10^{-3}$)					
	GA		Cohort Status		GA-cohort Interaction	
	β	p	β	p	β	p
Frontal	33.8	**<0.01***	487	**<0.01***	-16.9	**<0.01***
Parietal	54.6	**<0.01***	636	**<0.01***	-22.8	**<0.01***
Temporal	46.4	**<0.01***	481	**<0.01***	-16.7	**<0.01***
Occipital	46.7	**<0.01***	325	**<0.01***	-12.1	**<0.01***
	Sulcal Depth ($\times 10^{-3}$ mm)					
	GA		Cohort Status		GA-cohort Interaction	
	β	p	β	p	β	p
Frontal	103	**<0.01***	1587	**<0.01***	-51.6	**<0.01***
Parietal	194	**<0.01***	1792	**<0.01***	-59.1	**<0.01***
Temporal	127	**<0.01***	1435	**<0.01***	-45.0	**<0.01***
Occipital	153	**<0.01***	393	0.20	-15.3	0.12

Note: Bold p: p<0.05. *: q<0.05.

Supplementary Table 3. The results of the generalized estimating equations (GEEs) for the associations between fetal brain volumes/brain cortical features and cohort status (0: pre-pandemic; 1: pandemic), adjusting for gestational age at MRI (weeks) and fetal sex, by the two brain hemispheres.

	Brain volumes (mm^3)			
	Left Hemisphere		Right Hemisphere	
	β	p	β	p
CGM	-51	0.94	585	0.39
WM	-2534	**<0.01***	-3261	**<0.01***
DGM	-111	0.40	-137	0.28
Hippocampus	-37	**<0.01***	-42	**<0.01***
Cerebellum	-371	**<0.01***	-105	0.27
	Lobe Surface Area (mm^2)			
	Left Hemisphere		Right Hemisphere	
	β	p	β	p
Frontal	-243	**<0.01***	-228	**<0.01***
Parietal	-226	**<0.01***	-235	**<0.01***
Temporal	-197	**<0.01***	-244	**<0.01***
Occipital	-119	**<0.01***	-135	**<0.01***
	Local Gyrification Index ($\times 10^{-3}$)			
	Left Hemisphere		Right Hemisphere	
	β	p	β	p
Frontal	-69	**<0.01***	-65	**<0.01***
Parietal	-111	**<0.01***	-114	**<0.01***
Temporal	-46	**<0.01***	-92	**<0.01***
Occipital	-68	**<0.01***	-74	**<0.01***
	Sulcal Depth ($\times 10^{-3}$ mm)			
	Left Hemisphere		Right Hemisphere	
	β	p	β	p
Frontal	-157	**<0.01***	-55	0.32
Parietal	-150	**0.02***	-143	0.06
Temporal	49	0.32	-140	**0.02**
Occipital	-120	**0.01***	-95	**0.04**

Note: The β and p represent the coefficient and its significance of the cohort status in each GEE. CGM: Cortical gray matter. WM: White matter. DGM: Deep gray matter. Bold p: $p<0.05$. *: $q<0.05$.

Supplementary Table 4. The results of the generalized estimating equations (GEEs) for the associations between fetal brain volumes/brain cortical features and cohort status (0: pre-pandemic; 1: pandemic), adjusting for gestational age (GA) at MRI (weeks), fetal sex, and each of the parental education or employment.

	Brain volumes (mm^3)							
	Maternal Education		Paternal Education		Maternal Employment		Paternal Employment	
	β	p	β	p	β	p	β	p
CGM	-974	0.11	-466	0.29	-528	0.22	-634	0.11
WM	1324	0.24	2138	**0.01***	548	0.38	1318	0.13
DGM	123	0.32	172	0.08	-22	0.81	85	0.39
Hippocampus	11	0.32	8	0.33	6	0.32	3	0.67
Cerebellum	228	**0.02**	206	0.05	93	0.17	178	0.05
Brainstem	45	0.10	42	**0.04**	5	0.74	34	0.09
	Lobe Surface Area (mm^2)							
	Maternal Education		Paternal Education		Maternal Employment		Paternal Employment	
	β	p	β	p	β	p	β	p
Frontal	4.1	0.93	27.0	0.45	-6.7	0.86	-21.5	0.62
Parietal	-32.6	0.31	-14.4	0.57	-21.2	0.37	-32.5	0.14
Temporal	-18.6	0.55	3.7	0.87	-15.7	0.45	-24.8	0.31
Occipital	7.5	0.76	15.4	0.41	7.1	0.55	-2.5	0.89
	Local Gyrification Index ($\times 10^{-3}$)							
	Maternal Education		Paternal Education		Maternal Employment		Paternal Employment	
	β	p	β	p	β	p	β	p
Frontal	-7.6	0.24	-8.2	0.10	-6.7	0.07	-10.9	**0.03***
Parietal	-14.5	0.13	-16.8	**0.03**	-8.8	0.13	-16.5	**0.02***
Temporal	-11.4	0.12	-7.4	0.22	-7.7	0.13	-12.4	**0.01***
Occipital	-11.7	0.17	-14.6	**0.04**	-5.5	0.24	-11.0	0.06
	Sulcal Depth ($\times 10^{-3}$ mm)							
	Maternal Education		Paternal Education		Maternal Employment		Paternal Employment	
	β	p	β	p	β	p	β	p
Frontal	-8.2	0.74	-14.8	0.47	-0.5	0.97	-17.9	0.46
Parietal	-44.3	0.23	-46.8	0.10	-13.3	0.59	-41.9	0.20
Temporal	-30.0	0.24	-27.1	0.23	-16.1	0.40	-31.9	0.11
Occipital	-14.7	0.53	-40.2	**0.04**	-2.1	0.88	-25.6	0.15

Note: The β and p represent the coefficient and its significance of the parental education or employment in each GEE. CGM: Cortical gray matter. WM: White matter. DGM: Deep gray matter. Bold p: p<0.05. *: q<0.05.

Supplementary Table 5. Results of generalized estimating equations (GEEs) for the associations between maternal distress measures and cohort status (0: pre-pandemic; 1: pandemic), adjusting for gestational age at MRI (weeks) and fetal sex. The low and high mental distress groups were determined based on their corresponding threshold (SSAI: 40; STAI: 40; PSS: 15; EPDS: 10).

All Subjects							
	Pre-pandemic		Pandemic		β	95% CI	p
	N	LS Mean±SE	N	LS Mean±SE			
SSAI	163	29.4±2.8	72	31.0±3.2	1.59	[-1.23, 4.41]	0.27
STAI	161	30.9±2.5	71	32.9±2.9	1.99	[-0.68, 4.66]	0.14
PSS	160	10.5±1.8	74	14.4±2.0	3.92	[2.03, 5.81]	**<0.01**
EPDS	160	4.2±1.2	72	5.9±1.4	1.70	[0.50, 2.90]	**0.01**
Low Mental Distress Group							
	Pre-pandemic		Pandemic		β	95% CI	p
	N	LS Mean±SE	N	LS Mean±SE			
SSAI	146	27.1±2.1	59	27.6±2.2	0.51	[-1.14, 2.15]	0.55
STAI	136	27.9±1.7	60	30.2±1.9	2.36	[0.58, 4.14]	**0.01**
PSS	125	8.1±1.3	38	9.2±1.5	1.07	[-0.17, 2.31]	0.09
EPDS	147	3.4±0.9	60	4.6±1.0	1.23	[0.37, 2.09]	**0.01**
High Mental Distress Group							
	Pre-pandemic		Pandemic		β	95% CI	p
	N	LS Mean±SE	N	LS Mean±SE			
SSAI	17	48.5±5.5	13	47.0±6.2	-1.46	[-6.84, 3.92]	0.59
STAI	25	46.8±6.3	11	47.4±6.7	0.54	[-3.86, 4.95]	0.81
PSS	35	18.8±2.4	36	19.8±2.6	1.02	[-0.79, 2.82]	0.27
EPDS	13	13.1±3.5	12	12.6±3.7	-0.49	[-2.76, 1.78]	0.68

Note: The β and p represent the coefficient and its significance of cohort status in each GEE. N: Number of scans. SSAI: Spielberger State Anxiety Inventory. STAI: Spielberger Trait Anxiety Inventory. PSS: Perceived Stress Scale. EPDS: Edinburgh Postnatal Depression Scale. LS Mean: Least squares mean. SE: Standard error. CI: Confidence interval. Bold p: p<0.05.

Supplementary Table 6. The results of the generalized estimating equations (GEEs) for the associations between fetal brain volumes/brain cortical features and each maternal distress measure, adjusting for gestational age at MRI (weeks) and fetal sex for all subjects (including both pre-pandemic and pandemic cohorts).

	Brain volumes (mm^3)							
	SSAI		STAI		PSS		EPDS	
	β	p	β	p	β	p	β	p
CGM	54.6	0.40	46.4	0.49	29.6	0.75	180.2	0.22
WM	-71.4	0.43	-198.4	**0.03**	-226.5	0.10	-89.0	0.71
DGM	5.5	0.67	-6.2	0.63	-14.3	0.38	36.0	0.21
Hippocampus	-2.1	**0.02**	-3.0	**0.01***	-3.6	**0.02***	-4.7	0.06
Cerebellum	-16.7	0.08	-20.5	**0.03**	-42.0	**0.01***	-42.9	0.09
Brainstem	1.4	0.54	0.2	0.95	2.7	0.42	0.4	0.95
	Lobe Surface Area (mm^2)							
	SSAI		STAI		PSS		EPDS	
	β	p	β	p	β	p	β	p
Frontal	5.79	0.21	0.62	0.90	-3.02	0.69	7.82	0.54
Parietal	1.08	0.78	-1.14	0.76	-6.94	0.26	-1.80	0.86
Temporal	2.55	0.47	0.51	0.89	-4.06	0.41	2.11	0.82
Occipital	1.57	0.56	-1.05	0.70	-3.43	0.38	1.28	0.84
	Local Gyrification Index ($\times 10^{-3}$)							
	SSAI		STAI		PSS		EPDS	
	β	p	β	p	β	p	β	p
Frontal	0.55	0.36	0.32	0.63	-0.77	0.47	-0.50	0.74
Parietal	0.89	0.41	0.57	0.60	-0.95	0.59	-0.13	0.96
Temporal	1.47	0.10	1.45	0.13	0.84	0.48	2.96	0.19
Occipital	1.41	0.07	0.95	0.26	0.27	0.83	1.45	0.44
	Sulcal Depth ($\times 10^{-3}$ mm)							
	SSAI		STAI		PSS		EPDS	
	β	p	β	p	β	p	β	p
Frontal	3.12	0.18	3.57	0.16	1.69	0.63	6.86	0.25
Parietal	1.43	0.67	4.51	0.19	3.54	0.49	13.53	0.08
Temporal	5.71	**0.02**	6.04	**0.03**	6.54	0.07	13.60	**0.03**
Occipital	3.75	0.09	3.16	0.19	2.31	0.49	7.90	0.13

Note: The β and p represent the coefficient and its significance of each maternal distress measure in each GEE. SSAI: Spielberger State Anxiety Inventory. STAI: Spielberger Trait Anxiety Inventory. PSS: Perceived Stress Scale. EPDS: Edinburgh Postnatal Depression Scale. CGM: Cortical gray matter. WM: White matter. DGM: Deep gray matter. Bold p: p<0.05. *: q<0.05.

Supplementary Table 7. The results of sensitivity analysis considering MR scans with gestational age greater than/equal to 28 weeks. The results show associations between fetal brain volumes/brain cortical features and cohort status (0: pre-pandemic; 1: pandemic), adjusting for gestational age at MRI (weeks), fetal sex, and each maternal distress measure.

	Brain volumes (mm^3)							
	SSAI		STAI		PSS		EPDS	
	β	p	β	p	β	p	β	p
CGM	382	0.83	394	0.83	250	0.88	186	0.92
WM	-4363	0.06	-4122	0.07	-4742	0.06	-4716	0.06
DGM	408	0.06	411	0.06	321	0.16	279	0.21
Hippocampus	-89	**<0.01***	-87	**<0.01***	-82	**<0.01***	-86	**<0.01***
Cerebellum	-694	**<0.01***	-676	**<0.01***	-721	**<0.01***	-710	**<0.01***
Brainstem	30	0.61	25	0.68	0.38	0.99	3.28	0.96
	Lobe Surface Area (mm^2)							
	SSAI		STAI		PSS		EPDS	
	β	p	β	p	β	p	β	p
Frontal	-531	**<0.01***	-525	**<0.01***	-557	**<0.01***	-549	**<0.01***
Parietal	-513	**<0.01***	-513	**<0.01***	-530	**<0.01***	-530	**<0.01***
Temporal	-472	**<0.01***	-477	**<0.01***	-500	**<0.01***	-493	**<0.01***
Occipital	-286	**<0.01***	-283	**<0.01***	-308	**<0.01***	-302	**<0.01***
	Local Gyrification Index ($\times 10^{-3}$)							
	SSAI		STAI		PSS		EPDS	
	β	p	β	p	β	p	β	p
Frontal	-79	**<0.01***	-79	**<0.01***	-80	**<0.01***	-79	**<0.01***
Parietal	-130	**<0.01***	-130	**<0.01***	-132	**<0.01***	-131	**<0.01***
Temporal	-85	**<0.01***	-87	**<0.01***	-94	**<0.01***	-90	**<0.01***
Occipital	-79	**<0.01***	-80	**<0.01***	-84	**<0.01***	-81	**<0.01***
	Sulcal Depth ($\times 10^{-3}$ mm)							
	SSAI		STAI		PSS		EPDS	
	β	p	β	p	β	p	β	p
Frontal	-130	**0.03***	-137	**0.02***	-143	**0.02***	-137	**0.02***
Parietal	-178	**0.02***	-190	**0.01***	-212	**0.01***	-202	**0.01***
Temporal	-92	0.10	-103	0.08	-120	**0.04***	-112	0.05
Occipital	-123	**0.01***	-127	**0.01***	-137	**0.01***	-138	**0.01***

Note: The β and p represent the coefficient and its significance of the cohort status in each GEE. SSAI: Spielberger State Anxiety Inventory. STAI: Spielberger Trait Anxiety Inventory. PSS: Perceived Stress Scale. EPDS: Edinburgh Postnatal Depression Scale. CGM: Cortical gray matter. WM: White matter. DGM: Deep gray matter. Bold p: p<0.05. *: q<0.05.

Supplementary Table 8. The results of sensitivity analysis considering subjects with maternal age less than/equal to 40. The results show associations between fetal brain volumes/brain cortical features and cohort status (0: pre-pandemic; 1: pandemic), adjusting for gestational age at MRI (weeks), fetal sex, and each maternal distress measure.

	Brain volumes (mm^3)							
	SSAI		STAI		PSS		EPDS	
	β	p	β	p	β	p	β	p
CGM	843	0.55	903	0.52	901	0.51	489	0.73
WM	-5059	**0.01***	-4830	**0.01***	-5106	**0.01***	-5291	**0.01***
DGM	119	0.60	137	0.54	180	0.41	45	0.85
Hippocampus	-77	**<0.01***	-75	**<0.01***	-71	**<0.01***	-75	**<0.01***
Cerebellum	-474	**0.02***	-449	**0.03**	-436	**0.047**	-483	**0.03**
Brainstem	14	0.76	23	0.64	4.4	0.93	8.7	0.86
	Lobe Surface Area (mm^2)							
	SSAI		STAI		PSS		EPDS	
	β	p	β	p	β	p	β	p
Frontal	-415	**<0.01***	-407	**<0.01***	-431	**<0.01***	-438	**<0.01***
Parietal	-425	**<0.01***	-419	**<0.01***	-433	**<0.01***	-439	**<0.01***
Temporal	-396	**<0.01***	-395	**<0.01***	-413	**<0.01***	-412	**<0.01***
Occipital	-255	**<0.01***	-251	**<0.01***	-271	**<0.01***	-271	**<0.01***
	Local Gyrification Index ($\times10^{-3}$)							
	SSAI		STAI		PSS		EPDS	
	β	p	β	p	β	p	β	p
Frontal	-65	**<0.01***	-64	**<0.01***	-64	**<0.01***	-65	**<0.01***
Parietal	-113	**<0.01***	-111	**<0.01***	-112	**<0.01***	-114	**<0.01***
Temporal	-75	**<0.01***	-76	**<0.01***	-80	**<0.01***	-80	**<0.01***
Occipital	-75	**<0.01***	-74	**<0.01***	-77	**<0.01***	-76	**<0.01***
	Sulcal Depth ($\times10^{-3}$ mm)							
	SSAI		STAI		PSS		EPDS	
	β	p	β	p	β	p	β	p
Frontal	-93	0.10	-94	0.10	-96	0.11	-102	0.07
Parietal	-144	**0.047**	-150	**0.04**	-160	**0.04**	-170	**0.02***
Temporal	-77	0.15	-78	0.16	-91	0.10	-91	0.09
Occipital	-121	**0.01***	-120	**0.01***	-123	**0.02**	-135	**<0.01***

Note: The β and p represent the coefficient and its significance of the cohort status in each GEE. SSAI: Spielberger State Anxiety Inventory. STAI: Spielberger Trait Anxiety Inventory. PSS: Perceived Stress Scale. EPDS: Edinburgh Postnatal Depression Scale. CGM: Cortical gray matter. WM: White matter. DGM: Deep gray matter. Bold p: p<0.05. *: q<0.05.

Supplementary Figure 1. Flow diagram summarizing our subject recruitment in this study.

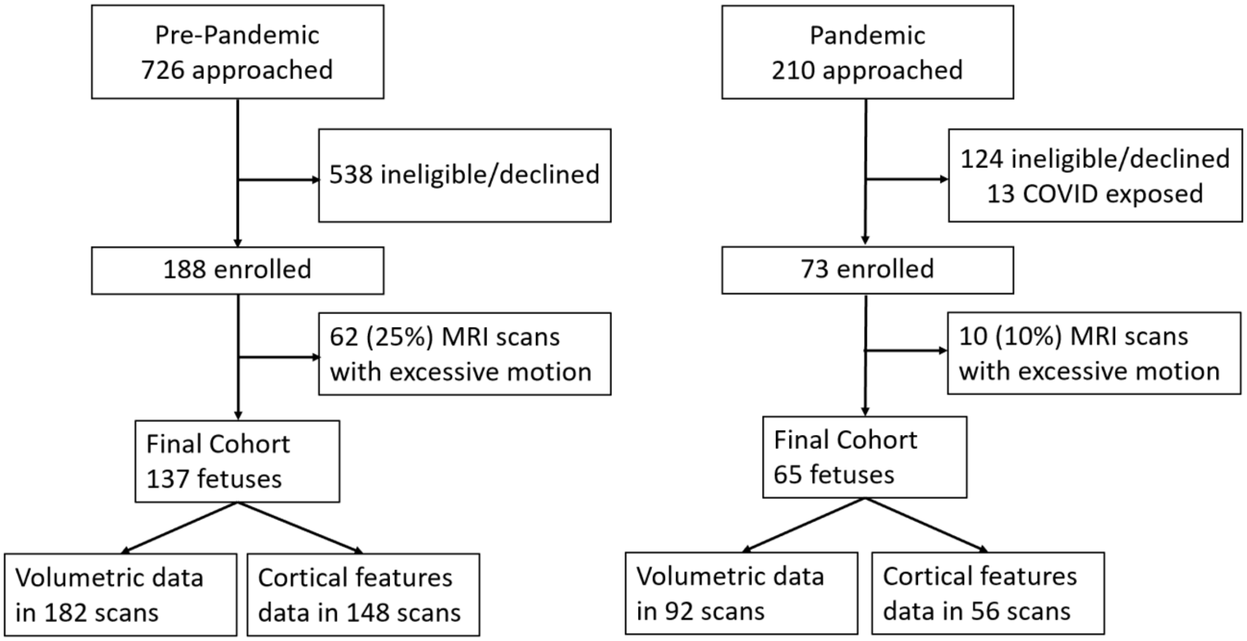

Eligible pregnant women were recruited from community maternal fetal medicine offices and were referred by their obstetrics providers. The study team spoke to potential participants alongside the obstetricians and would follow up with those interested in participation. Written informed consent was obtained from all participants before completing study procedures.

Supplementary Figure 2. Illustration of brain tissue segmentation and parcellation of a male fetus with gestational age of 33.3 weeks.

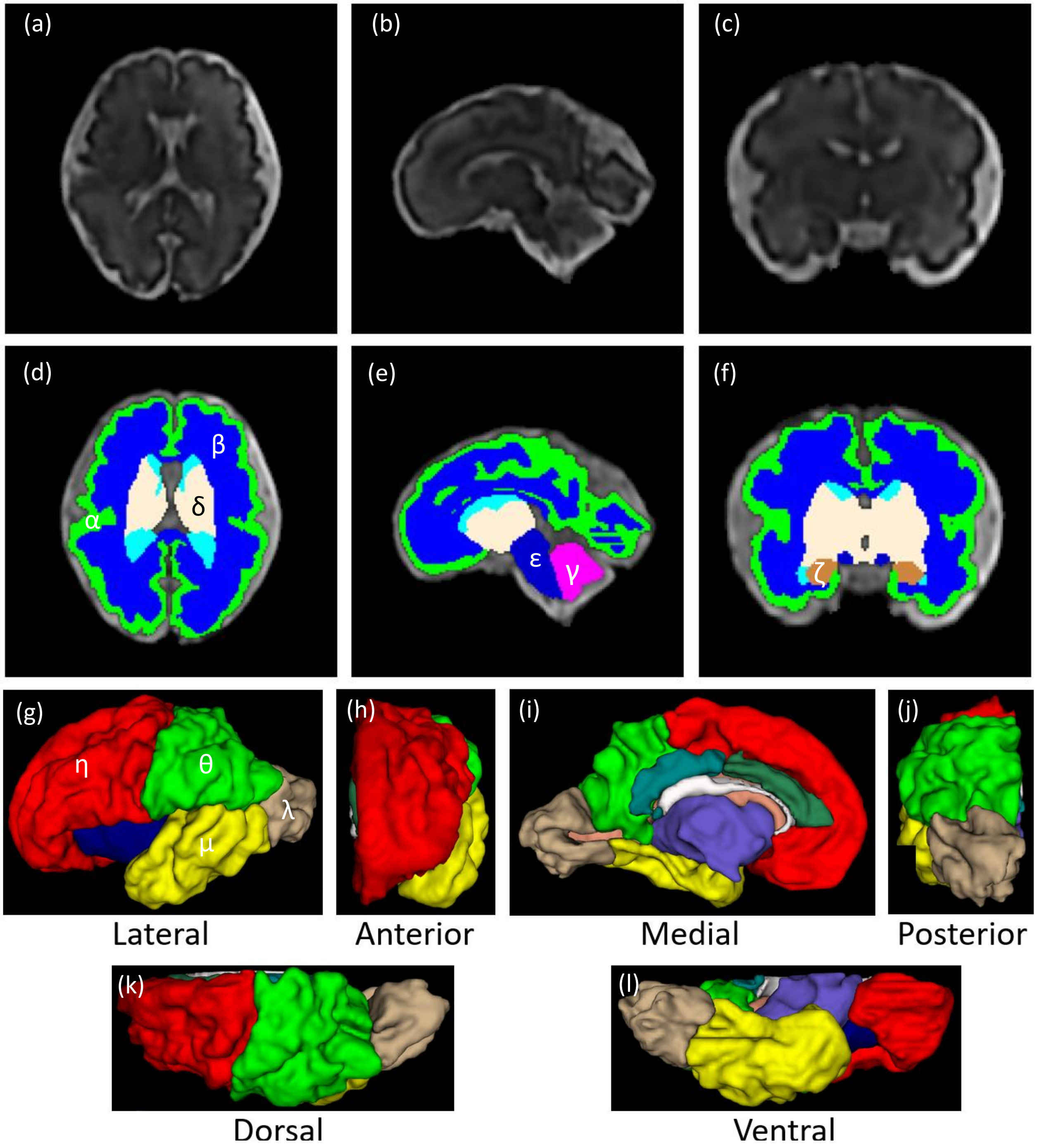

a-c: T2-weighted images, where a: axial plane, b: sagittal plane, and c: coronal plane. d-f: Corresponding image segmentation. g-l: Parcellation of the left hemisphere. α: Cortical gray matter. β: White matter. γ: Cerebellum. δ: Deep gray matter. ε: Brainstem. ζ: Hippocampus. η: Frontal. θ: Parietal. μ: Temporal. λ: Occipital.

Supplementary Figure 3. The distribution of dates that the included fetal scans were completed.

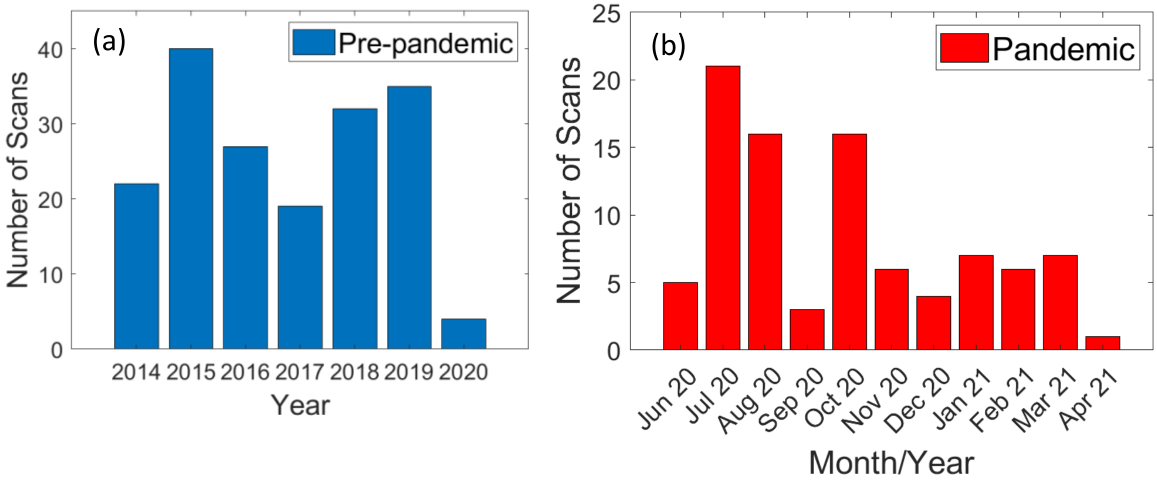

(a) Pre-pandemic cohort; (b) pandemic cohort.
