# Supplementary material for: Maternal psychological distress during the COVID-19 pandemic and structural changes of the human fetal brain
Source: Commun Med (Lond). 2022 May 26;2:47. doi: 10.1038/s43856-022-00111-w (PMC9135751; doi:10.1038/s43856-022-00111-w)
Supplement: Supplementary file 4 — Description of Additional Supplementary Files [file 43856_2022_111_MOESM4_ESM.pdf]

Description of Additional Supplementary Files

File Name: Supplementary Data 1

Description: Supplementary Data for Figure 1

File Name: Supplementary Data 2

Description: Supplementary Data for Figure 2
