# Supplementary material for: Maternal psychological distress during the COVID-19 pandemic and structural changes of the human fetal brain
Source: Commun Med (Lond). 2022 May 26;2:47. doi: 10.1038/s43856-022-00111-w (PMC9135751; doi:10.1038/s43856-022-00111-w)
Supplement: Supplementary file 5 — Reporting Summary [file 43856_2022_111_MOESM5_ESM.pdf]

nature research

Corresponding author(s): DBPR COMMSMED-21-0443A

Last updated by author(s): Apr 6, 2022

Reporting Summary

Nature Research wishes to improve the reproducibility of the work that we publish. This form provides structure for consistency and transparency in reporting. For further information on Nature Research policies, see our Editorial Policies and the Editorial Policy Checklist.

Statistics

For all statistical analyses, confirm that the following items are present in the figure legend, table legend, main text, or Methods section.

n/a	Confirmed	
☐	☒	The exact sample size (n) for each experimental group/condition, given as a discrete number and unit of measurement
☐	☒	A statement on whether measurements were taken from distinct samples or whether the same sample was measured repeatedly
☐	☒	The statistical test(s) used AND whether they are one- or two-sided<br>*Only common tests should be described solely by name; describe more complex techniques in the Methods section.*
☐	☒	A description of all covariates tested
☐	☒	A description of any assumptions or corrections, such as tests of normality and adjustment for multiple comparisons
☐	☒	A full description of the statistical parameters including central tendency (e.g. means) or other basic estimates (e.g. regression coefficient) AND variation (e.g. standard deviation) or associated estimates of uncertainty (e.g. confidence intervals)
☐	☒	For null hypothesis testing, the test statistic (e.g. F, t, r) with confidence intervals, effect sizes, degrees of freedom and P value noted<br>*Give P values as exact values whenever suitable.*
☒	☐	For Bayesian analysis, information on the choice of priors and Markov chain Monte Carlo settings
☒	☐	For hierarchical and complex designs, identification of the appropriate level for tests and full reporting of outcomes
☒	☐	Estimates of effect sizes (e.g. Cohen's d, Pearson's r), indicating how they were calculated

Our web collection on statistics for biologists contains articles on many of the points above.

Software and code

Policy information about availability of computer code

Data collection	1.5 Tesla GE Discovery MR450 scanner (GE Healthcare, Milwaukee, WI, USA)
Data analysis	ITK-SNAP 3.8.0, MATLAB R2019a (The MathWorks, Inc., Natick, MA, USA)

For manuscripts utilizing custom algorithms or software that are central to the research but not yet described in published literature, software must be made available to editors and reviewers. We strongly encourage code deposition in a community repository (e.g. GitHub). See the Nature Research guidelines for submitting code & software for further information.

Data

Policy information about availability of data

All manuscripts must include a data availability statement. This statement should provide the following information, where applicable:
- Accession codes, unique identifiers, or web links for publicly available datasets
- A list of figures that have associated raw data
- A description of any restrictions on data availability

The datasets generated and/or analyzed during the current study are available from the corresponding author on reasonable request.

Field-specific reporting

Please select the one below that is the best fit for your research. If you are not sure, read the appropriate sections before making your selection.

☒ Life sciences ☐ Behavioural & social sciences ☐ Ecological, evolutionary & environmental sciences

For a reference copy of the document with all sections, see nature.com/documents/nr-reporting-summary-flat.pdf

Life sciences study design

All studies must disclose on these points even when the disclosure is negative.

Sample size

202 pregnant women

Data exclusions

Exclusion criteria were multiple gestation pregnancy, known or suspected congenital infection, syndromic or dysgenetic features in the fetus, documented chromosomal abnormalities, or any maternal contraindication to MRI. Enrolled fetuses found to have structural (encephaloclastic or dysgenetic) brain abnormalities on fetal MRI, or postnatal confirmation of a genetic syndrome were subsequently excluded from the study.

Replication

Subjects were scanned up to 2 time points during pregnancy. Standard MRI procedure was applied to each scan. No replication was conducted in each scan according to the IRB protocol.

Randomization

Controlled covariates include gestational age at scan, fetal sex, and maternal distress measures (Spielberger State Anxiety Inventory, Spielberger Trait Anxiety Inventory, Perceived Stress Scale, and Edinburgh Postnatal Depression Scale.)

Blinding

Brain segmentations were manually corrected by trained research team members, who were blinded to pandemic vs. pre-pandemic cohort status.

Reporting for specific materials, systems and methods

We require information from authors about some types of materials, experimental systems and methods used in many studies. Here, indicate whether each material, system or method listed is relevant to your study. If you are not sure if a list item applies to your research, read the appropriate section before selecting a response.

Materials & experimental systems

n/a	Involved in the study	
☒	☐	Antibodies
☒	☐	Eukaryotic cell lines
☒	☐	Palaeontology and archaeology
☒	☐	Animals and other organisms
☐	☒	Human research participants
☐	☒	Clinical data
☒	☐	Dual use research of concern

Methods

n/a	Involved in the study	
☒	☐	ChIP-seq
☒	☐	Flow cytometry
☐	☒	MRI-based neuroimaging

Human research participants

Policy information about studies involving human research participants

Population characteristics

The final data set consisted of 202 pregnant women (pre-pandemic: 137; pandemic: 65) between 16.7 to 39.1 gestational weeks, in which a total of 274 fetal brain MRI scans were acquired. Seventy (26%) MRI scans (pre-pandemic: 34; pandemic: 36) failed brain surface reconstruction and therefore were not used for cortical folding calculations. Among the 202 study participants, 72 participants were scanned twice during pregnancy (45 pre-pandemic and 27 pandemic) while all other subjects were scanned once (92 pre-pandemic and 38 pandemic). The median GA at MRI was 30.2 weeks (range: 16.7 to 39.1) for the pre-pandemic group and was 30.8 weeks (range: 17 to 38.4) for the pandemic group. The median maternal age for the entire cohort was 33.9 years old (range: 17 to 51). The median GA at birth was 39.6 weeks (range: 31.0 to 41.9), and the median birth weight was 3.36 kg (range: 1.02 to 4.70).

Recruitment

This study involved two sequential enrollments: (1) 137 healthy pregnant women from March 2014 to February 2020 ("pre-pandemic"); (2) 65 women without confirmed COVID-19 exposures from June 2020 to April 2021 ("pandemic") for a fetal brain magnetic resonance imaging (MRI) study from low-risk obstetrical community hospitals in Washington, DC. The first enrollment period was part of a longitudinal study of normative fetal brain development in low-risk obstetric patients, and the second was a natural history observational study of fetal brain development during the COVID-19 pandemic. Study procedures were identical across both enrollment periods.

Ethics oversight

This study was approved by the local Institutional Review Board. Written informed consent was obtained from all

Ethics oversight	participants.

Note that full information on the approval of the study protocol must also be provided in the manuscript.

Clinical data

Policy information about clinical studies

All manuscripts should comply with the ICMJE guidelines for publication of clinical research and a completed CONSORT checklist must be included with all submissions.

Clinical trial registration	Not applicable
Study protocol	Protocol 1373 and Protocol 14257
Data collection	We enrolled low-risk and health women during two distinct epochs 137 healthy pregnant women from March 2014 to February 2020 (“pre-pandemic”) and 65 women without confirmed COVID-19 exposures from June 2020 to April 2021 from low-risk obstetrical community hospitals in Washington, DC.
Outcomes	Maternal distress measures, fetal brain measures of regional and global volumes and surface analyses

Magnetic resonance imaging

Experimental design

Design type	Prospective cohort study
Design specifications	Each scan was limited within 2 hours per subject. Each subject was scanned up to two time points in the fetal period.
Behavioral performance measures	Multi-plane multi-phase single shot fast spin echo (SSFSE) T2-weighted images for fetal brain were acquired. Four well-validated maternal distress measures were completed by each pregnant woman on the day of the MRI, including Spielberger State Anxiety Inventory, Spielberger Trait Anxiety Inventory, Perceived Stress Scale, and Edinburgh Postnatal Depression Scale.

Acquisition

Imaging type(s)	Structural
Field strength	1.5 Tesla
Sequence & imaging parameters	The following acquisition parameters were used: echo time = 160 ms; repetition time = 1100 ms; field of view = 320 × 320 mm^2; matrix = 256 × 256; 2 mm slice thickness and 50 to 70 consecutive slices for full fetal brain coverage in the axial, coronal, and sagittal planes for a final in-plane resolution of 1.25 × 1.25 mm^2.
Area of acquisition	Whole fetal brain

Diffusion MRI ☐ Used ☒ Not used

Preprocessing

Preprocessing software	Automatic segmentation of the brain tissues was then implemented using the Developing Brain Region Annotation with Expectation-Maximization (Draw-EM) algorithm. Manual correction of tissue labels of the segmentation and parcellation files was performed by a trained research team member using ITK-SNAP.
Normalization	The Draw-EM pipeline did not implement normalization on target images. This was done to preserve the structural information in the 3D MRI.
Normalization template	The Draw-EM pipeline did not implement normalization on target images. The segmented atlas was registered to the target image and the atlas images were propagated to the image.
Noise and artifact removal	Volume reconstruction using Kainz's approach was utilized for noise and artifact removal.<br>Kainz B, Steinberger M, Wein W, et al. Fast volume reconstruction from motion corrupted stacks of 2D slices. IEEE Trans Med Imaging. 2015;34(9):1901-1913.
Volume censoring	The Draw-EM pipeline did not implement censoring on target images.

Statistical modeling & inference

Model type and settings	Univariate analysis, Shapiro-Wilks test, generalized estimating equations
Effect(s) tested	For the generalized estimating equations, we tested the effect of the parameters of cohort status (0: pre-pandemic; 1: pandemic), adjusting for gestational age at MRI (weeks), fetal sex, and each maternal distress measure.

Specify type of analysis: ☐ Whole brain ☒ ROI-based ☐ Both

Anatomical location(s): Automatic segmentation of the brain tissues was implemented using the Developing Brain Region Annotation with Expectation-Maximization (Draw-EM) algorithm. The regions include cortical gray matter, white matter, deep gray matter, hippocampus, cerebellum, and brainstem. The cortical features were calculated based on the outer surface of the white matter.

Statistic type for inference (See Eklund et al. 2016): This study was not involving fMRI, so this study was not affected by the issues raised in Eklund et al. 2016.

Correction: All p values were adjusted for multiple testing using the false discovery rate method based on the number of outcomes (6 tissues or 4 lobes).

Models & analysis

n/a	Involved in the study
☒	☐ Functional and/or effective connectivity
☒	☐ Graph analysis
☐	☒ Multivariate modeling or predictive analysis

Multivariate modeling and predictive analysis: The response variables were fetal brain regional volumes and cortical features. The main predictor was the pandemic status (pre-pandemic: 0 [referent]; pandemic: 1). The independent variables were fetal gestational age, fetal sex, and maternal distress measures.
